# Social Determinants of Health and Life Satisfaction of Children With Disabilities

**DOI:** 10.1111/cch.70175

**Published:** 2025-11-14

**Authors:** Sinyoung Choi

**Affiliations:** ^1^ College of Nursing Seoul National University Seoul Republic of Korea

**Keywords:** children with disabilities, psychological well‐being, quality of life, social determinants of health

## Abstract

**Background:**

Creating a supportive environment for the health and well‐being of children with disabilities is essential to enhancing their quality of life and promoting positive outcomes for families and society. In South Korea, children with disabilities face numerous structural and social challenges that threaten their well‐being and lower their life satisfaction. This study aimed to examine the factors affecting life satisfaction among school‐attending children with disabilities aged 19 years or younger in South Korea, using a framework based on the social determinants of health.

**Methods:**

A secondary data analysis was conducted using the 2022 Panel Survey of Disability and Life Dynamics by the Korea Disabled People's Development Institute. A total of 569 children were included. Variables were selected based on the five domains of the Healthy People 2030 framework: economic stability, education access and quality, health care access and quality, neighbourhood and built environment and social and community context. Hierarchical regression analysis was used to determine the influence of these factors on life satisfaction.

**Results:**

Children with disabilities in South Korea reported lower life satisfaction than other vulnerable groups, which also face constraints on health and well‐being due to socioeconomic, cultural or environmental factors. Significant predictors of life satisfaction were functional limitations, parental depression, type of education, experience with learning accommodations, satisfaction with disability welfare services, family strength, peer attachment, subjective health and self‐esteem.

**Conclusions:**

This study highlights the importance of addressing the social determinants of health that shape the life satisfaction of children with disabilities in South Korea. Future research should broaden the scope to include diverse populations and contexts, thereby informing more inclusive and tailored strategies to promote their health and well‐being.

## Summary

- Children with disabilities in South Korea are likely to experience reduced life satisfaction due to constraints within social determinants of health, which may threaten their psychosocial health and overall quality of life.
- Significant predictors of life satisfaction were functional limitations, parental depression, type of education, learning accommodations, satisfaction with disability welfare services, family strength, peer attachment, subjective health and self‐esteem.
- Findings highlight the importance for an integrated approach that considers both individual and social determinants in understanding life satisfaction among children with disabilities.
- Future research should include a broader population of children with disabilities and prioritize self‐reports to ensure that their voices are systematically incorporated into policy and practice.

## 1 Introduction

In 2023, the number of children with disabilities under the age of 18 in South Korea was approximately 90 000, accounting for about 1.2% of the total child population (Ministry of Health and Welfare 2023). Although this is a relatively small number compared to other age groups with disabilities, the proportion has continued to rise steadily in recent years (Kim et al. 2023). Considering children's developmental growth and life‐course trajectory, childhood is a critically important period for establishing the foundation of a healthy and fulfilling life across the lifespan (Kim and Kim 2015).

Appropriate healthcare and well‐being support during childhood can prevent the worsening of disabilities and promote psychosocial stability, thereby enabling children with disabilities to develop self‐confidence and build positive social relationships (Hamdani et al. 2018). Children with disabilities who experience a healthy childhood are more likely to reach their full potential and lead productive and independent lives in adulthood, ultimately helping to lessen long‐term social and economic costs (Coleman et al. 2022). Furthermore, their health and well‐being directly contribute to alleviating family stress, strengthening household stability and enhancing overall quality of life. Greater societal attention to these issues also plays a critical role in improving public understanding of disability, thereby fostering a more inclusive and resilient society (Hamdani et al. 2018; Majnemer et al. 2007). Thus, ensuring the health and well‐being of children with disabilities is essential not only for their individual happiness but also for the collective health, social cohesion and sustainable development of society.

Life satisfaction is a multidimensional and complex construct that encompasses all aspects of human life, including physical, psychological, emotional and social dimensions (Coleman et al. 2022; Proctor et al. 2017). The term is often used interchangeably with related concepts such as well‐being, happiness and quality of life (Diener 1984; Jun et al. 2015). According to the World Health Organization's International Classification of Functioning, Disability and Health for Children and Youth (ICF‐CY), health conditions, body functions and structures, activities and participation and environmental factors interact dynamically in children with disabilities. Understanding this interrelated framework is essential for supporting their well‐being and promoting healthy development (World Health Organization 2007).

Historically, children with disabilities have experienced substantial health disparities compared with their non‐disabled peers, largely due to their impairments (Shilling et al. 2012). These disparities have led to adverse outcomes in overall well‐being and life satisfaction (Savage et al. 2014). According to the International Survey of Children's Well‐Being, a standardized international comparative study on children's quality of life, children with disabilities report lower levels of life satisfaction than their non‐disabled counterparts. This disparity reflects not only intrinsic factors but also adverse socio‐environmental conditions (Coleman et al. 2022; Save the Children 2021). Given that South Korea ranked 31st out of 35 countries in children's overall happiness (Gwyther Rees et al. 2020), life satisfaction among children with disabilities in the country may be particularly low. While research on children's life satisfaction has expanded in recent years, studies specifically focusing on children with disabilities remain scarce. Furthermore, many have a narrow scope or rely heavily on proxy responses instead of directly engaging the children themselves (Hamdani et al. 2018; Savage et al. 2014; Save the Children 2021).

The social determinants of health (SDOH) refer to ‘the conditions in which people are born, grow, live, work and age’ (World Health Organization 2010). They are widely recognized as a core strategy for addressing health inequalities both between and within countries, offering an established approach to promoting health equity, particularly among vulnerable populations (World Health Organization 2010). This framework emphasizes the influence of social, economic and environmental factors on health and well‐being, providing a comprehensive lens through which to understand disparities in life satisfaction among children with disabilities (Majnemer et al. 2007).

Schools serve as a primary microsystem and key socialization context in which children develop their identities and form attitudes toward present and future life. Accordingly, understanding life satisfaction among school‐aged children with disabilities is essential not only for ensuring their rights and well‐being but also for building a more inclusive and equitable society (Bronfenbrenner 1992; Maciver et al. 2019). Therefore, this study aimed to identify the SDOH among children with disabilities under the age of 19 who are enrolled in elementary, middle or high schools and to comprehensively examine the factors influencing their life satisfaction.

## 2 Methods

### 2.1 Study Design

This study was a secondary data analysis aimed at evaluating the factors influencing life satisfaction among children with disabilities aged 19 years or younger who wereenrolled in elementary, middle or high schools.

### 2.2 Data and Sample

This study utilized raw data from the fifth wave (2022) of the Disability and Life Dynamics Panel (DLDP), conducted by the Korea Disabled People's Development Institute (KODDI) and approved as official national statistics by the Korean government (National Statistics Approval No. 438001). The DLDP is a nationally representative survey of community‐dwelling individuals with disabilities, covering domains such as disability acceptance, health and medical care, independent living and social participation. The respondents included individuals with disabilities (the ‘panel’), as well as household heads and members aged 12 years or older. If direct responses from a panel member were not feasible, proxy responses were permitted from the person who shared the most daily life with them (e.g., a family or household member).

The DLDP sample was drawn using a two‐stage sampling design. In the first stage, districts (eup, myeon and dong) were allocated priority if they included residents with rare disabilities or individuals aged 18 years or younger, to ensure their inclusion in the final sample. Rare disabilities were defined as low‐prevalence types, including speech disability, autism spectrum disorder, cardiac disability, respiratory disability, hepatic disability, facial disability, ostomy‐related disability and epilepsy‐related disability. After this prioritization, probability proportional systematic sampling was applied, taking regional distribution into account. In the second stage, individuals within the selected districts were stratified by type of disability, severity and gender, and systematic sampling was subsequently conducted within each stratum. Data were collected through face‐to‐face interviews by trained interviewers using Tablet‐Assisted Personal Interviewing (Korea Disabled People's Development Institute 2024).

**FIGURE 1:**
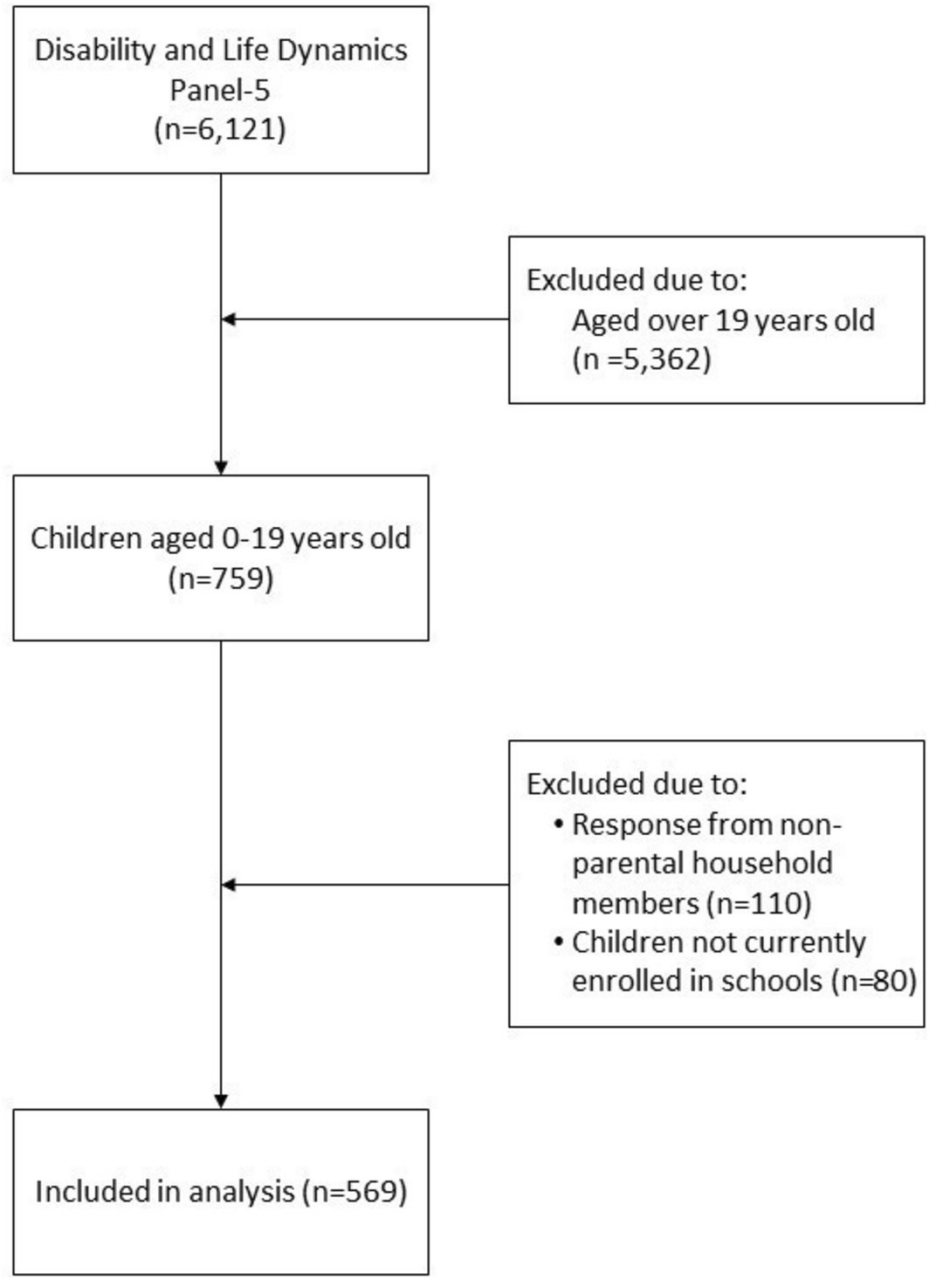
Flow diagram of study participants.

For this study, the analytic sample consisted of school‐aged children (aged 19 years or younger) who were enrolled in elementary, middle or high schools and participated in the fifth wave of the DLDP. Of the 759 eligible participants, a final sample of 569 cases was selected in which a parent (father or mother) was identified as a household member. This selection was based on the assumption that the parent, as the primary caregiver, plays a central role in shaping the child's life satisfaction (Figure 1). Although the DLDP employed a complex sampling design to ensure national representativeness, sampling weights were not applied because this study focused on a specific subpopulation, namely school‐aged children with disabilities whose primary caregiver was a parent.

### 2.3 Theoretical Framework

This study applied the SDOH framework proposed by Healthy People 2030, a U.S. national initiative for public health promotion and disease prevention. Healthy People 2030 identifies five key domains of SDOH: economic stability; education access and quality; health care access and quality; neighbourhood and built environment; and social and community context. These domains guide strategic objectives to create healthier environments and reduce health disparities (Office of Disease Prevention and Health Promotion 2020). Guided by this framework, variables were selected based on their conceptual fit within the five SDOH domains and their relevance to the study's objectives (Appendix 1 and Supporting Information).

### 2.4 Variables

#### 2.4.1 General Characteristics

The general characteristics of children with disabilities included gender, age, household income, school level, type and severity of primary disability, presence of multiple disabilities and functional limitations. Functional limitations were assessed using a 14‐item scale developed by the KODDI, based on the Australian Survey of Disability, Ageing and Carers (SDAC). In the DLDP, disability‐related information was collected based on the primary disability recorded on the Korean Disability Registration Card, the official government‐issued identification for persons with disabilities in South Korea. This card allows up to two types of disabilities to be recorded in order of severity. Respondents with two recorded disabilities were defined as having multiple disabilities. The severity of the primary disability was categorized as ‘severe’ (grades 1–3) or ‘mild’ (grades 4–6), according to the six‐level disability grading system formerly used in South Korea until June 2019. Because the DLDP commenced in 2018, respondents reported severity as ‘severe’ or ‘mild’, with explicit guidance on correspondence to the previous six‐level grading system to ensure consistency across waves.

#### 2.4.2 Parental Characteristics

The parental characteristics were included in recognition of the critical role that parents play in the health and well‐being of children with disabilities. Variables included gender, age, marital status, educational level and disability status, as well as psychological variables such as subjective health, perceived stress, depression and self‐esteem. Depression and self‐esteem were assessed using the Korean 11‐item Center for Epidemiologic Studies Depression Scale (K‐CES‐D‐11) and the Korean Rosenberg Self‐Esteem Scale (K‐RSES), respectively.

#### 2.4.3 Health Status

The health status of children with disabilities was assessed using four indicators: subjective health, perceived stress, depression and self‐esteem. Subjective health was measured with a single item (‘How would you rate your overall health over the past six months?’), and perceived stress with a single item (‘How much stress do you usually feel in daily life?’); both were rated on a 4‐point scale. Depression and self‐esteem were measured using the same instruments used for the parents.

#### 2.4.4 SDOH

##### 2.4.4.1 Economic Stability

Economic stability was operationalized as the burden of healthcare expenses, which was evaluated using a single item (‘What is the most burdensome household expense?’). Responses were recoded as ‘high’ if healthcare was identified as the primary burden, and ‘low’ if another expense was selected or if no burden was reported.

##### 2.4.4.2 Education Access and Quality

This domain included school commute time, type of education and experience with learning accommodations. School commute time was categorized into four groups: less than 20, 20–39, 40–59 and 60 min or more. Type of education was classified as general or special education, according to the school setting. Experience with learning accommodations was assessed with five items: (1) provision of school transportation; (2) appropriate installation or modification of restrooms; (3) installation or adaptation of ramps for mobility; (4) availability of learning or communication support devices (e.g., height‐adjustable desks, braille materials, hearing aids); and (5) provision of support personnel for school life and learning. Responses were recoded as ‘yes’ if at least one form of support was reported and ‘no’ if none were reported.

##### 2.4.4.3 Health Care Access and Quality

Access to health care was assessed based on walking time to the nearest medical institution and recoded into two categories: less than 30 and 30 min or more. Health care quality was evaluated with four items: (1) healthcare providers' understanding of disability and treatment; (2) sufficiency of medical result explanations; (3) satisfaction with medical services; and (4) satisfaction with disability welfare services. Each item was rated on a 4‐point Likert scale, with higher scores indicating more favourable perceptions.

##### 2.4.4.4 Neighbourhood and Built Environment

Neighbourhood and built environment were assessed using an 11‐item scale developed by the KODDI, comprising three subdomains: (1) housing structure, performance and physical environment; (2) convenience of the surrounding neighbourhood; and (3) perceived residential stability. Each item was rated on a 4‐point Likert scale, with higher scores reflecting more favourable and healthier living conditions.

##### 2.4.4.5 Social and Community Context

Social and community context included family strength, peer attachment and perceived respect in daily life. Family strength was evaluated using a 20‐item scale developed by Eo and Yoo (1995) and revised by Choi (2004). Peer attachment was assessed using a 9‐item version of the Inventory of Parent and Peer Attachment (IPPA), adapted for the Korean context. Perceived social respect was assessed with a single item asking how much respect the respondent felt from others in daily life, rated on a 4‐point scale.

#### 2.4.5 Life Satisfaction

Life satisfaction was measured using an instrument developed by the KODDI, which includes eight domains: health, income, residential environment, school life, employment, marital life, social relationships and overall life satisfaction. Each item was rated on a 10‐point Likert scale. In the DLDP, items related to income, employment and marital life were administered only to respondents aged 19 and older. Given the study population's age range and the potential conceptual overlap with other variables, only the item on overall life satisfaction was used for analysis.

### 2.5 Data Analysis

Data were analysed using IBM SPSS Statistics version 30.0 (IBM Corp., Armonk, NY, USA), with a two‐tailed significance level set at 0.05. Descriptive statistics, including frequencies, percentages, means and standard deviations, were calculated to summarize the general characteristics of children with disabilities, their parents, health status, SDOH and life satisfaction. Group differences in life satisfaction were examined using independent *t*‐tests, Mann–Whitney *U* tests and one‐way ANOVA, as appropriate. Pearson correlation coefficients were calculated to explore associations among the key variables. Finally, hierarchical regression analysis was conducted to identify factors influencing life satisfaction among children with disabilities.

## 3 Results

### 3.1 General Characteristics and Health Status of Children With Disabilities and Their Parents

Among the children, 368 (64.7%) were in their teenage years and 347 (61.0%) were male. The majority were enrolled in elementary school (77.9%) (Table 1). The most frequently reported primary type of disability was hearing or speech disability (34.8%), followed by intellectual disability or autism spectrum disorder (30.1%). A total of 361 children (63.4%) had severe disabilities, and 19.2% had multiple disabilities. A total of 240 (42.2%) had a rare disability, with speech disability constituting the largest proportion (21.8%). The mean subjective health score over the past six months was 2.97 ± 0.48. The mean scores for perceived stress, depression and self‐esteem were 2.01 ± 0.70, 4.92 ± 5.32 and 28.64 ± 3.89, respectively.

**TABLE 1:** Differences and correlations in life satisfaction according to general characteristics and health status of children with disabilities (*N* = 569).

Characteristics	Categories	*n* (%)	Life satisfaction	
			Mean ± SD	T/F/r (*p*)
**General characteristics**				
Gender	Male	347 (61.0)	6.42 ± 1.97	−1.09 (0.275)
	Female	222 (39.0)	6.60 ± 1.86	
Age (year)	< 10	201 (35.3)	6.57 ± 2.04	0.76 (0.446)
	10–19	368 (64.7)	6.44 ± 1.87	
Monthly household income (KRW)	< 2 million	102 (17.9)	6.48 ± 1.71	1.28 (0.279)
	2–4 million	327 (57.5)	6.55 ± 1.95	
	4–6 million	118 (20.7)	6.24 ± 2.08	
	≥ 6 million	22 (3.9)	7.00 ± 1.72	
School level	Elementary school	443 (77.9)	6.49 ± 1.95	0.08 (0.935)
	Middle/high school	126 (22.1)	6.48 ± 1.88	
Type of primary disability	Physical disability	47 (8.3)	6.19 ± 2.13	0.96 (0.445)
	Brain lesion disability	83 (14.6)	6.35 ± 2.06	
	Visual disability	36 (6.3)	6.75 ± 1.81	
	Hearing/speech disability	198 (34.8)	6.67 ± 1.91	
	Intellectual disability/ASD	171 (30.1)	6.36 ± 1.86	
	Internal organ/facial disability^a^	34 (6.0)	6.59 ± 1.91	
Severity of primary disability	Severe	361 (63.4)	6.37 ± 1.99	−1.92 (0.056)
	Mild	208 (36.6)	6.69 ± 1.81	
Multiple disability	Yes	109 (19.2)	6.20 ± 2.21	−1.61 (0.107)^b^
	No	460 (80.8)	6.56 ± 1.86	
Functional limitations			24.17 ± 7.61	−0.300 (< 0.001)
**Health status**				
Subjective health			2.97 ± 0.48	0.408 (< 0.001)
Perceived stress			2.01 ± 0.70	−0.238 (< 0.001)
Depression			4.92 ± 5.32	−0.365 (< 0.001)
Self‐esteem			28.64 ± 3.89	0.284 (< 0.001)
Life satisfaction			6.49 ± 1.93	

^a^ Internal organ/facial disability: facial, cardiac, respiratory, hepatic and renal disabilities, as well as ostomy‐related disabilities (including colostomy, ileostomy and urostomy) and epilepsy‐related disabilities.

^b^ Mann–Whitney test.

Abbreviations: ASD = autism spectrum disorder; SD = standard deviation.

Among the parents, 312 (54.8%) were female, and the majority were in their 40s (366 individuals, 64.3%). Regarding marital status, 90.9% were living with a spouse and 64.5% had completed a university‐level education or higher. Seventeen parents (3.0%) were registered as having a disability. The mean scores for subjective health, perceived stress, depression and self‐esteem were 2.89 ± 0.37, 2.33 ± 0.63, 5.49 ± 5.53 and 30.00 ± 3.57, respectively.

### 3.2 SDOH of Children With Disabilities

In households with children with disabilities, 15.6% of respondents reported medical expenses as their primary household financial burden (Table 2). With respect to education type, 264 children (46.4%) were enrolled in general classes at regular schools, while 305 children (53.6%) attended special classes in regular schools, special education schools or transition programs. The mean scores for family strength and peer attachment were 71.26 ± 7.86 and 23.75 ± 3.68, respectively.

**TABLE 2:** Differences and correlations in life satisfaction according to social determinants of health (*N* = 569).

Variables	Categories	*n* (%)	Life satisfaction	
			Mean ± SD	T/F/r (*p*)
**Economic stability**				
Burden of healthcare expenses	High	89 (15.6)	5.81 ± 2.09	−3.65 (< 0.001)
	Low	480 (84.4)	6.61 ± 1.88	
**Education access and quality**				
Type of education	General	264 (46.4)	6.69 ± 1.75	2.36 (0.019)
	Special	305 (53.6)	6.31 ± 2.06	
School commute time (minutes)	< 20	131 (23.0)	6.74 ± 1.91	1.38 (0.248)
	20–39	330 (58.0)	6.36 ± 1.90	
	40–59	63 (11.1)	6.65 ± 2.13	
	≥ 60	45 (7.9)	6.47 ± 1.90	
Experience with learning accommodations	Yes	239 (42.0)	6.02 ± 2.08	−5.02 (< 0.001)
	No	330 (58.0)	6.83 ± 1.75	
**Health care access and quality**				
Walking time to medical institutions (minutes)	< 30	463 (81.4)	6.60 ± 1.89	2.79 (0.005)
	≥ 30	106 (18.6)	6.02 ± 2.05	
Healthcare providers' understanding of disability and treatment			3.03 ± 0.45	0.077 (0.066)
Sufficiency of medical result explanation			3.05 ± 0.46	0.021 (0.625)
Satisfaction with medical services			2.97 ± 0.30	0.093 (0.027)
Satisfaction with disability welfare services			2.70 ± 0.53	0.218 (< 0.001)
**Neighbourhood and built environment**				
Living environment			34.43 ± 4.68	0.226 (< 0.001)
**Social and community context**				
Family strength			71.26 ± 7.86	0.259 (< 0.001)
Peer attachment			23.75 ± 3.68	0.193 (< 0.001)
Perceived social respect			2.80 ± 0.49	0.227 (< 0.001)

Abbreviation: SD = standard deviation.

### 3.3 Relationships Between General and Parental Characteristics, Health Status and Life Satisfaction of Children With Disabilities

Life satisfaction among children with disabilities showed significant negative correlations with the degree of limitation in daily life due to disability (*r* = −0.300, *p* < 0.001), perceived stress (*r* = −0.238, *p* < 0.001), depression (*r* = −0.365, *p* < 0.001), parental perceived stress (*r* = −0.269, *p* < 0.001) and parental depression (*r* = −0.364, *p* < 0.001). In contrast, it showed significant positive correlations with their subjective health (*r* = 0.408, *p* < 0.001), self‐esteem (*r* = 0.284, *p* < 0.001), parental subjective health (*r* = 0.201, *p* < 0.001) and parental self‐esteem (*r* = 0.259, *p* < 0.001).

### 3.4 Differences in and Relationships Between Life Satisfaction and SDOH in Children With Disabilities

Life satisfaction among children with disabilities significantly differed according to SDOH, including the burden of healthcare expenses, type of education, experience with learning accommodations and walking time to medical institutions. Children from households reporting a higher burden of healthcare expenses reported significantly lower life satisfaction than those with a lower burden (*t* = −3.65, *p* < 0.001). Similarly, children in special education reported lower life satisfaction than those in general education (*t* = 2.36, *p* = 0.019). In contrast, children without experience of learning accommodations showed higher life satisfaction than those with such experience (*t* = −5.02, *p* < 0.001), and children within a 30‐min walking distance of medical institutions reported higher life satisfaction than those farther away (*t* = 2.79, *p* = 0.005). Furthermore, life satisfaction was positively correlated with satisfaction with medical services (*r* = 0.093, *p* = 0.027), satisfaction with disability welfare services (*r* = 0.218, *p* < 0.001), living environment (*r* = 0.226, *p* < 0.001), family strength (*r* = 0.259, *p* < 0.001), peer attachment (*r* = 0.193, *p* < 0.001) and perceived social respect (*r* = 0.227, *p* < 0.001).

### 3.5 Factors Influencing the Life Satisfaction of Children With Disabilities

To identify the factors influencing life satisfaction among children with disabilities, hierarchical regression analysis was conducted using variables that showed significant differences or correlations with children's and parents' characteristics, health status and SDOH. The analysis was performed in three steps. Based on the premise that the SDOH constitute the fundamental social, economic and environmental conditions that shape individual health and well‐being (Office of Disease Prevention and Health Promotion 2020), the independent variables were sequentially entered as follows.

In Step 1, child and parental characteristics were entered: functional limitations, parental subjective health, perceived stress, depression and self‐esteem. In Step 2, SDOH were added: burden of healthcare expenses, type of education, experience with learning accommodations, walking time to medical institutions, satisfaction with medical services, satisfaction with disability welfare services, living environment, family strength, peer attachment and perceived social respect. In Step 3, child health status variables were added: subjective health, perceived stress, depression and self‐esteem. Assumptions for regression analysis were verified using residual scatterplots and normal probability (P–P) plots, confirming residual normality and homoscedasticity. The Durbin–Watson statistic was 2.08, indicating independence of residuals. Multicollinearity was not a concern, with tolerance values ranging from 0.39 to 0.95 and variance inflation factors ranging from 1.05 to 2.55.

The results of the hierarchical regression analysis indicated that Model 1, which included child and parental characteristics, was statistically significant (*F* = 28.18, *p* < 0.001), accounting for 19% of the variance in life satisfaction (Table 3). Model 2, which added SDOH, was also significant (*F* = 15.34, *p* < 0.001) and accounted for 28% of the variance, an additional 9% over Model 1. Finally, Model 3, which included health status variables, was statistically significant (*F* = 15.73, *p* < 0.001), increasing the explained variance to 33%. In the final model, significant predictors of life satisfaction included subjective health of the child (*β* = 0.23, *p* < 0.001), self‐esteem (*β* = 0.09, *p* = 0.023), functional limitation (*β* = −0.09, *p* = 0.024), parental depression (*β* = −0.13, *p* = 0.023), type of education (*β* = −0.09, *p* = 0.025), experience with learning accommodations (*β* = −0.10, *p* = 0.010), satisfaction with disability welfare services (*β* = 0.13, *p* < 0.001), family strength (*β* = 0.09, *p* = 0.021) and peer attachment (*β* = 0.08, *p* = 0.047).

**TABLE 3:** Hierarchical multiple regression model for life satisfaction of children with disabilities.

Variables	Model 1					Model 2					Model 3				
	*B*	SE	*β*	*t*	*p*	*B*	SE	*β*	*t*	*p*	B	SE	*β*	*t*	*p*
**Child and parental characteristics**															
Functional limitations	−0.05	0.01	−0.21	−5.42	< 0.001	−0.04	0.01	−0.17	−4.23	< 0.001	−0.02	0.01	−0.09	−2.27	0.024
Parental subjective health	0.42	0.21	0.08	1.99	0.047	0.27	0.20	0.05	1.36	0.174	0.10	0.20	0.02	0.50	0.615
Parental perceived stress	−0.30	0.13	−0.10	−2.27	0.023	−0.16	0.13	−0.05	−1.28	0.200	−0.16	0.13	−0.05	−1.24	0.216
Parental depression	−0.08	0.02	−0.21	−4.51	< 0.001	−0.06	0.02	−0.18	−3.71	< 0.001	−0.04	0.02	−0.13	−2.29	0.023
Parental self‐esteem	0.03	0.02	0.06	1.42	0.157	0.03	0.02	0.05	1.09	0.275	0.01	0.02	0.02	0.37	0.716
**Social determinants of health**															
Burden of healthcare expenses (Ref: low)						−0.31	0.20	−0.06	−1.56	0.119	−0.17	0.19	−0.03	−0.90	0.371
Type of education (Ref: general)						−0.36	0.16	−0.09	−2.29	0.023	−0.34	0.15	−0.09	−2.25	0.025
Experience with learning accommodations (Ref: No)						−0.45	0.16	−0.11	−2.88	0.004	−0.39	0.15	−0.10	−2.60	0.010
Walking time to medical institutions (Ref: < 30 min)						−0.18	0.18	−0.04	−0.98	0.329	−0.22	0.18	−0.04	−1.24	0.217
Satisfaction with medical services						−0.33	0.24	−0.05	−1.37	0.172	−0.33	0.24	−0.05	−1.39	0.165
Satisfactions with disability welfare services						0.49	0.14	0.14	3.56	< 0.001	0.48	0.13	0.13	3.57	< 0.001
Living environment						0.02	0.02	0.06	1.37	0.171	0.01	0.02	0.03	0.81	0.418
Family strength						0.03	0.01	0.13	3.18	0.002	0.02	0.01	0.09	2.32	0.021
Peer attachment						0.04	0.02	0.08	1.95	0.051	0.04	0.02	0.08	1.99	0.047
Perceived social respect						0.41	0.15	0.10	2.64	0.009	0.28	0.15	0.07	1.86	0.064
**Health status**															
Subjective health											0.93	0.15	0.23	6.04	< 0.001
Perceived stress											−0.07	0.11	−0.02	−0.60	0.550
Depression											−0.02	0.02	−0.05	−0.91	0.361
Self‐esteem											0.05	0.02	0.09	2.28	0.023
*F* (*p*)	28.18 (< 0.001)					15.34 (< 0.001)					15.73 (< 0.001)				
*R* ^2^	0.20					0.29					0.35				
Adj. *R* ^2^	0.19					0.28					0.33				

## 4 Discussion

In this study, the mean life satisfaction score among children with disabilities was 6.49 (out of 10). Although differences in measurement instruments limit direct comparability, this result is broadly consistent with international findings. For example, scores were 56.56 (out of 100) in Hong Kong (Yang et al. 2022), 69.25 (out of 100) in Singapore (Kan et al. 2025) and 39.42 (out of 50) in Portugal (Gaspar et al. 2016). In contrast, studies on other vulnerable populations in South Korea reported higher levels of life satisfaction, including 8.53 and 8.15 among elementary and middle school students attending community child centres (Jo 2021) and 8.13 among multicultural adolescents (Sohn 2023) (all out of 10). Taken together, the findings of this study suggest that life satisfaction among children with disabilities is a shared concern across diverse contexts. Moreover, the findings indicate that these children may face greater challenges to their quality of life, even in comparison with other vulnerable groups whose opportunities for health and well‐being are constrained by socioeconomic, cultural or environmental factors.

Life satisfaction is a key component of individual happiness and well‐being. The World Happiness Report 2019 by the United Nations warned of growing global inequality in happiness stemming from disparities in opportunity (Helliwell et al. 2019). For children with disabilities, who often face personal, social and cultural discrimination alongside structural inequalities, life satisfaction is especially salient (Choi and Kim 2014). Therefore, it is crucial to examine the specific factors that may further reduce life satisfaction among children with disabilities compared to other vulnerable groups. Based on these insights, tailored strategies and interventions should be developed and implemented to promote their well‐being.

The results of the hierarchical regression analysis revealed a progressive increase in explanatory power for life satisfaction as additional variables were included. The notable observation in Step 2, with the inclusion of SDOH, suggests that life satisfaction in this population is shaped not only by individual characteristics but also by broader social and environmental factors. The further increase in Step 3, after the addition of children's health status variables, demonstrates that psychosocial factors, such as subjective health, perceived stress, depression and self‐esteem, also play a critical role in explaining life satisfaction alongside SDOH. These findings highlight the importance of developing comprehensive intervention strategies that not only address structural and environmental barriers through social support systems but also incorporate targeted approaches to promote psychosocial well‐being among children with disabilities.

This study found that children with disabilities who had used learning support accommodations reported lower levels of life satisfaction compared to those who had not. In South Korea, the ‘Anti‐Discrimination against and Remedies for Persons with Disabilities Act’ stipulates educational accommodations as a legitimate right, including transportation support for commuting, accessible facilities within schools, the allocation of support personnel, assistive devices for learning and communication, appropriate teaching and assessment methods and the establishment of disability support departments (Ministry of Government Legislation 2023). Based on this legal and institutional framework, this study operationalized ‘learning accommodations’ as encompassing mobility and facility improvements, supports for learning and communication and personal assistance. However, prior research has indicated that, in practice, accommodations in educational settings have often been limited to physical aspects, whereas direct academic and human supports for learning remain insufficient. Moreover, studies have pointed out that such accommodations often fail to address disability‐specific needs and lack consistency due to non‐standardized procedures (Hong et al. 2022; Ok et al. 2022). Within this context, when accommodations do not effectively meet the actual needs of children with disabilities, their utilization may not necessarily lead to improved life satisfaction.

Furthermore, the provision of learning accommodations may be accompanied by unintended social stigma. Qualitative research with students with disabilities in South Korea showed that, even when accommodations provide meaningful academic assistance, students often experienced stigma, discomfort and burden due to peers' and teachers' limited disability awareness and insufficient understanding of the significance of such support. The visible use of assistive devices, in particular, highlighted differences from peers, leading some students to avoid necessary supports for fear of ‘appearing different’ (Ok et al. 2022). Similarly, international studies have found that students with disabilities have experienced stigma and labelling related to their need for learning support, which undermines self‐esteem and hinders psychological adjustment (Haft et al. 2023; Shifrer 2013). While the degree of stigma may vary across sociocultural contexts, in South Korea, where public awareness and acceptance of disability remain in a transitional stage, accommodations may be perceived less as legitimate rights and more as privileges or markers of difference (Kim et al. 2023; Ok et al. 2022).

Accordingly, improving learning accommodations requires a multidimensional approach. This entails not only strengthening physical and academic support, but also actively reducing stigma and labelling, enhancing public recognition of accommodations as rights, developing tailored supports to address disability‐specific needs and establishing standardized procedures. In addition, fostering a socially and emotionally supportive school environment that encourages positive interactions between peers and teachers is critical for enabling children with disabilities to engage actively in learning and to develop a positive self‐concept. Such an integrated approach can serve as a foundation for promoting the overall development and social participation of children with disabilities and, ultimately, for enhancing their life satisfaction.

This study has several limitations. First, as life satisfaction is a multidimensional construct, additional unexamined factors may also influence the life satisfaction of children with disabilities. In particular, physical health variables were not considered. Physical health is a key determinant of quality of life that affects child development both directly and indirectly through interactions with environmental factors (Bronfenbrenner 1992). Future research should incorporate physical health indicators to provide a more comprehensive understanding of the relationship between health status and life satisfaction in children with disabilities. Second, to enable consistent assessment across the five key domains of SDOH, this study excluded children who were not attending school for reasons such as medical treatment, rehabilitation, concerns about stigma and prejudice or other contextual barriers. As a result, these children could not respond to survey items related to educational access and quality. Consequently, the findings may not be generalizable to all children with disabilities, particularly those not currently enrolled in school, and should be interpreted with caution. Third, the original dataset did not disclose whether responses were self‐reported by the child or proxy‐reported, making it impossible to verify this information. According to the survey design, however, all responses for children under the age of 10 were collected through proxy reporting, accounting for 35.3% of the sample. This substantial proportion of proxy responses may introduce potential bias, and caution is warranted when interpreting the findings.

Nevertheless, this study is significant in that it investigated the multidimensional factors influencing life satisfaction among children with disabilities within the SDOH framework, which is globally recognized as a core public health strategy. This integration of individual‐level and socio‐environmental variables allowed the study to systematically identify key determinants of life satisfaction in this population. These findings provide valuable evidence to inform the development of interventions aimed at reducing health disparities and promoting well‐being among children with disabilities. These may include enhancing children's self‐concepts and psychological health, family‐centred practices that support both child development and parental well‐being and peer‐based approaches that foster social inclusion. Furthermore, these findings may serve as a foundation for the formulation of targeted policies and supportive systems to advance equitable health outcomes.

## 5 Conclusion

This study examined the SDOH and life satisfaction among school‐attending children with disabilities under the age of 19 and identified the key factors influencing their life satisfaction. The findings revealed that children with disabilities reported lower levels of life satisfaction than other vulnerable populations, suggesting that their psychosocial health and overall well‐being may be at risk. Significant influencing factors included functional limitations, parental depression, type of education, experience with learning accommodations, satisfaction with disability welfare services, family strength, peer attachment, subjective health and self‐esteem.

Children with disabilities are often underrepresented or excluded from mainstream society due to a combination of factors such as age, social status, limited autonomy, communication difficulties and the type and severity of their disabilities. In this context, the present study is significant because it included children in South Korea with a wide range of disabilities, including rare conditions. This approach provides a more comprehensive and in‐depth understanding of life satisfaction among children with disabilities.

Based on the findings and discussion of this study, the following recommendations are proposed. First, to deepen understanding of the SDOH and life satisfaction among children with disabilities, foundational data should be accumulated through repeated studies using diverse approaches, including qualitative, mixed‐methods and longitudinal designs. Such approaches may provide insights into contextual and temporal dynamics that shape their life satisfaction. Second, children with disabilities who are unable to attend school may face distinct challenges, including limited opportunities for peer interaction, restricted access to school‐based resources and greater health‐related burdens, which could affect their life satisfaction in ways not captured in this study. Future research should broaden the study population to include these groups to identify additional determinants of life satisfaction and inform the development of more inclusive and tailored support strategies. Third, greater emphasis should be placed on incorporating the voices of children with disabilities. Future studies should strengthen the use of self‐reports by employing age‐appropriate instruments, simplified response formats and communication aids to more accurately capture children's perspectives and ensure their systematic integration into research and policy.

## Author Contributions

The author was responsible for all aspects of this work, including the conception and design of the study, acquisition and analysis of data, interpretation of findings and drafting and revising the manuscript.

## Ethics Statement

This study was approved for review exemption by the Institutional Review Board of the Seoul National University (IRB No. E2505/004–009).

## Conflicts of Interests

The author declares no conflicts of interest.

## Supporting information


**Appendix 2.** Measurement Instruments.

## Data Availability

The data that support the findings of this study are available upon request from the Korea Disabled People's Development Institute (KODDI) Disability Statistics Data Portal (https://koddi.or.kr/stat/html/user/main/main).

## Variable Source Mapping for Merged Panel–Household Head–Household Member Data in the DLDP 5th Wave

**Table:**

Variables	Resource
**General characteristics**	
Gender	Panel
Age	Panel
Monthly household income	Household head
School level	Panel
Type of primary disability	Panel
Severity of primary disability	Panel
Multiple disability	Panel
Functional limitations	Panel
**Parental characteristics**	
Gender	Household member
Age	Household member
Marital status	Household member
Educational level	Household member
Disability status	Household member
Subjective health status	Household member
Perceived stress	Household member
Depression	Household member
Self‐esteem	Household member
**Health status of children**	
Subjective health status	Panel
Perceived stress	Panel
Depression	Panel
Self‐esteem	Panel
**Social determinants of health**	
Burden of healthcare expenses	Household head
Type of education	Panel
School commute time (minutes)	Panel
Experience with learning accommodations	Panel
Walking time to medical institutions (minutes)	Panel
Healthcare providers' understanding of disability and treatment	Panel
Sufficiency of medical result explanation	Panel
Satisfaction with medical services	Panel
Satisfaction with disability welfare services	Panel
Living environment	Panel
Family strength	Panel
Peer attachment	Panel
Perceived social respect	Panel
Life satisfaction	Panel

- Panel: Individuals with disabilities, as defined in Article 2 of the Korean Act on Welfare of Persons with Disabilities, who were registered under Article 32 of the same Act between 1 January 2015, and 31 December 2017, excluding those residing in disability residential facilities.
- Household head: The person primarily responsible for the household's livelihood, regardless of their legal relationship to the household.
- Household member: A person who has lived together and shared a household for at least 6 months, excluding paid employees (e.g., personal care assistants, housekeepers, drivers) and nonrelatives. However, cohabiting partners without legal marriage and other non‐relatives living together are included as household members.
