# Supplementary material for: Social Determinants of Health and Life Satisfaction of Children With Disabilities
Source: Child Care Health Dev. 2025 Nov 14;51(6):e70175. doi: 10.1111/cch.70175 (PMC12616770; doi:10.1111/cch.70175)
Supplement: Supplementary file 1 — Appendix 2. Measurement Instruments. [file CCH-51-e70175-s001.docx]

# **Appendix 2. Measurement Instruments**

## **2.4.1 General characteristics**

The general characteristics of children with disabilities included gender, age, household income, school level, type of primary disability, severity of primary disability, presence of multiple disabilities, and functional limitations. Functional limitations were assessed using a 14-item scale developed by the KODDI (Korea Disabled People's Development Institute), referencing the Australian Survey of Disability, Ageing and Carers (SDAC). Each item was rated on a 4-point Likert scale (1 = not at all, 4 = very much), with higher scores indicating greater limitations in daily life due to disability. Cronbach’s α in this study was .86.

## **2.4.2 Parental characteristics**

Parents constitute the most immediate environment—the microsystem—for children and serve as a fundamental context for their growth and development (Bronfenbrenner, 1992). For children with disabilities in particular, parents play a critical role in supporting health and quality of life by providing direct and consistent environmental support. Accordingly, this study included parental characteristics that may affect the life satisfaction of children with disabilities. Demographic variables included gender, age, marital status, educational level, and disability status. Psychological variables included subjective health status, perceived stress, depression, and self-esteem. Parental age was recategorized as follows: 39 or younger, 40s, 50s, and 60 or older (including those in their 60s and 70s). Educational level was classified into two categories: high school or lower (no education, elementary, middle, high school) and college or higher (associate degree, university, master’s, or doctoral degree).

## **2.4.3 Health status**

The health status of children with disabilities was examined using four variables: subjective health status, perceived stress, depression, and self-esteem. Subjective health status was evaluated using a single item: “How would you rate your overall health over the past six months?”, scored on a 4-point scale (1 = very poor, 4 = very good). Perceived stress was assessed using the item: “How much stress do you usually feel in daily life?”, scored on a 4-point scale (1 = very much, 4 = hardly at all). Depression was analyzed using the Korean version of the short-form CES-D (Center for Epidemiologic Studies Depression Scale), originally developed by Radloff (1977) and shortened to 11 items by Kohout et al. (1993). The scale consists of 11 items (two reverse-coded) rated on a 4-point Likert scale (0 = rarely or none of the time, 3 = most or all of the time), with higher scores indicating greater depressive symptoms. Cronbach’s α was .91 in Hoe et al. (2017), and .89 in the present study for both children and parents. Self-esteem was evaluated using the Korean version of the Rosenberg Self-Esteem Scale (K-RSES), developed by Rosenberg (1965) and translated by The 10-item scale includes five positive and five negative items, each rated on a 4-point Likert scale (1 = strongly disagree, 4 = strongly agree), with higher scores reflecting greater self-esteem. According to Lee et al. (2009), the Cronbach’s α was .81 for elementary school students, .85 for middle school students, and .83 for high school students. In the present study, Cronbach’s α was .77 for children and .71 for parents.

## **2.4.4 Social determinants of health**

**Economic stability**

Economic stability included the burden of healthcare expenses, based on a single survey item asking, “What is the most burdensome household expense?”. Responses were recoded as “high” if health care was identified as the primary burden, and “low” if another expense was selected or no burden was reported.

**Education access and quality**

Education access and quality included average daily commuting time to and from school, type of education, and experience with learning accommodations. The DLDP (Disability and Life Dynamics Panel) collected responses on average commuting time in 10-minute intervals, which were recoded into four categories for analysis: “less than 20 minutes,” “20 to less than 40 minutes,” “40 to less than 60 minutes,” and “60 minutes or more.” Type of education was reclassified based on school setting as either general education (general classroom in regular school) or special education (special class in regular school, special school, or transition program). Experience with learning accommodations was assessed using five items: (1) provision of school transportation, (2) appropriate installation or modification of restrooms, (3) installation or adaptation of ramps for mobility, (4) availability of assistive learning or communication devices (e.g., height-adjustable desks, braille materials, hearing aids), and (5) provision of assistants for school life and learning. Responses were recoded as “yes” if the respondent reported using at least one of these supports and "no" if none were used.

**Health care access and quality**

Health care access was assessed based on travel time to medical institutions such as clinics, hospitals, and public health centers. Responses were recategorized into two groups: “less than 30 minutes on foot” (including less than 5 minutes, 5 to less than 10 minutes, and 10 to less than 30 minutes) and “30 minutes or more on foot.” Health care quality included four indicators: healthcare providers’ understanding of disability and Treatment, Sufficiency of medical result explanation, satisfaction with medical services, and satisfaction with disability welfare services. Understanding of disability and treatment was measured by the item, “Do the doctors or nurses at the medical institution you usually visit have a good understanding of your disability and treatment methods?” Sufficiency of explanation was assessed by the item, “After the medical examination, did the doctor or nurse explain the results to you clearly and in sufficient detail?” Each of these two items were rated on a 4-point scale (1 = not at all, 2 = not really, 3 = somewhat, and 4 = very much). Satisfaction with medical services and disability welfare services was also rated on a 4-point scale (1 = very dissatisfied, 2 = somewhat dissatisfied, 3 = somewhat satisfied, and 4 = very satisfied).

**Neighborhood and built environment**

Neighborhood and built environment was assessed using a housing environment scale developed by the KODDI, based on national standards such as the Minimum Housing Standards, the National Survey on Persons with Disabilities, and the Korea Welfare Panel Study. The instrument consists of 11 items covering three subdomains: housing structure, performance, and physical environment (6 items, including one reverse-coded); convenience of the surrounding neighborhood (4 items); and perceived residential stability (1 item). All items were rated on a 4-point Likert scale (1 = strongly disagree, 4 = strongly agree), with higher scores indicating a healthier residential environment. In the present study, Cronbach’s α was .71.

**Social and community context**

Social and community context included family strength, peer attachment, and perceived social respect in daily life. Family strength was derived from a scale originally developed by Eo and Yoo (1995) and revised by Choi (2004). The instrument consists of 20 items rated on a 4-point Likert scale (1 = not at all, 4 = always), with higher scores indicating greater family strength. In a prior study (Choi, 2002), Cronbach’s α = .96; in the present study, it was .93. Peer attachment was assessed using a modified version of the Inventory of Parent and Peer Attachment (IPPA), developed Armsden and Greenberg (1987). The adapted scale consists of 9 items (including 3 reverse-coded items) rated on a 4-point Likert scale (1 = strongly disagree, 4 = strongly agree), with higher scores indicating stronger peer attachment. This instrument has been used in the Korea Child and Youth Panel Survey (KCYPS), where the Cronbach’s α was .83 (Wave 5); in the present study, it was .71. Perceived social respect in daily life was based on a single item: “As a person with a disability, to what extent do you feel respected in your daily life by those around you, such as family, friends, or neighbors?” Responses were rated on a 4-point scale (1 = not at all respected, 4 = highly respected), with higher scores indicating a stronger perception of respect.

## **2.4.5 Life satisfaction**

Life satisfaction was assessed using a scale developed by the KODDI, based on instruments from the Korea Welfare Panel Study, the Korean Labor and Income Panel Study, and the Panel Survey of Employment for the Disabled. The instrument includes eight items, each representing a domain of life satisfaction: health, income, residential environment, school life, employment, marital life, social relationships, and overall life satisfaction. Each item is rated on a 10-point Likert scale (1 = very dissatisfied, 10 = very satisfied), with higher scores indicating greater satisfaction in the respective domain. In the DLDP, the items on income, employment, and marital life were administered only to respondents aged 19 and older. Considering the age range of the study population and potential conceptual overlap, this study used only the single item on overall life satisfaction for analysis.

## **References**

Armsden, G. C., & Greenberg, M. T. (1987). The inventory of parent and peer attachment: Individual differences and their relationship to psychological well-being in adolescence. *Journal of Youth and Adolescence*, *16*(5), 427-454.

Bronfenbrenner, U. (1992). Ecological systems theory. In R. Vasta (Ed.), *Six theories of child development: Revised formulations and current issues* (pp. 187-249). Jessica Kingsley Publishers.

Choi, J.-H. (2002). Family stress and coping strategies among commuting couples. *Journal of Families and Better Life*, *22*(2), 69-83.

Choi, J.-H. (2004). A Study on the Family Stress and Coping Strategy, Family Strengths among Commuting Couples. *Journal of Families and Better Life*, *22*(2), 69-83.

Eo, E. J., & Yoo, Y. J. (1995). A Study on Development of the Scale for Measuring Family Strengths. *Journal of Families and Better Life*, 13(1), 145-156.

Hoe, M., Lee, S. H., & Kim, Y. S. (2017). Developing the Korean Version of the 11-item CES-DC. *Mental Health and Social Work*, *45*(1), 255-285. https://doi.org/10.24301/MHSW.2017.03.45.1.255

Kohout, F. J., Berkman, L. F., Evans, D. A., & Cornoni-Huntley, J. (1993). Two shorter forms of the CES-D (Center for Epidemiological Studies Depression) depression symptoms index. *Journal of Aging and Health*, *5*(2), 179-193. https://doi.org/10.1177/089826439300500202

Lee, J., Nam, S., Lee, M.-k., Lee, J.-h., & Lee, S. M. (2009). Rosenberg’ Self-Esteem Scale: Analysis of Item-Level Validity. *Korean Journal Of Counseling And Psychotherapy*, *21*(1), 173-189.

Radloff, L. S. (1977). The CES-D Scale: A self-report depression scale for research in the general population. *Applied Psychological Measurement*, *1*(3), 385-401. https://doi.org/10.1177/014662167700100306

Rosenberg, M. (1965). *Society and the Adolescent Self-Image*. Princeton University Press. http://www.jstor.org/stable/j.ctt183pjjh
